# Analysis of Plastic Deformation Instabilities at Elevated Temperatures in Hot-Rolled Medium-Mn Steel

**DOI:** 10.3390/ma12244184

**Published:** 2019-12-12

**Authors:** Aleksandra Kozłowska, Barbara Grzegorczyk, Marcin Staszuk, Paweł M. Nuckowski, Adam Grajcar

**Affiliations:** Department of Engineering Materials and Biomaterials, Silesian University of Technology, 18A Konarskiego Street, 44-100 Gliwice, Poland; aleksandra.kozlowska@polsl.pl (A.K.); barbara.grzegorczyk@polsl.pl (B.G.); marcin.staszuk@polsl.pl (M.S.); pawel.nuckowski@polsl.pl (P.M.N.)

**Keywords:** medium-Mn steel, Portevin–Le Chatelier phenomenon (PLC), elevated temperature, Transformation Induced Plasticity (TRIP) effect, retained austenite

## Abstract

The study addressed the microstructure and mechanical properties of hot-rolled advanced high-strength medium manganese steel. Some of the curves that were obtained in static tensile tests at deformation temperatures of 20–200 °C showed the occurrence of the heterogeneous plastic deformation phenomenon, called the Portevin-Le Chatelier (PLC) effect. The deformation temperature significantly influenced a serration character. The correlations between the deformation temperature, serration range, microstructural features, and fracture behavior were investigated. The curves showed no Lüders elongation as a result of the thermomechanical processing applied. The serrated flow phenomenon was observed at 60 and 140 °C. The serration type was different and the most enhanced at 140 °C, where the PLC effect was present in both uniform and post-uniform elongation ranges. The disappearance of serrations at 200 °C was related to the increased diffusion intensity.

## 1. Introduction

Advanced steels for automotive industry should provide the favorable combination of mechanical and technological properties, such as high strength, good formability, and weldability [1,2,3]. Nowadays, the medium-Mn Transformation Induced Plasticity (TRIP) steels that belong to the 3rd generation of Advanced High Strength Steels (AHSS) are of particular interest due to the higher strength and ductility than first generation TRIP steels. At the same time, leaner Mn alloying results in lower production costs in comparison to high-Mn Twinning Induced Plasticity (TWIP) steels. Most of studies concerning medium-Mn sheet steel are focused on production aspects. However, factors affecting the processing of steel sheets are rarely analyzed [1,4,5].

It was noted in the literature that medium-Mn steels are prone to a plastic instability phenomenon related to Lüders or Portevin–Le Chatelier (PLC) band formation [6,7,8]. Both types of instabilities may lead to the occurrence of numerous cracks during forming processes or delayed cracking after deep drawing [9]. Medium-Mn steels that are produced by cold-rolling and subsequent intercritical annealing may show a discontinuous yielding behavior [5,7,10]. However, in the case of hot-rolled grades, this effect does not occur [11,12]. It is related to the presence of mobile dislocations generated during previous hot-rolling or thermomechanical processing. The PLC bands are represented as characteristic serrations on the stress-strain curves. The occurrence of the PLC bands is much more irregular and it can be observed as various serration types in comparison to the Lüders bands [7,10]. The PLC effect in medium-Mn steels that is discussed in the literature is referenced to the dynamic strain ageing (DSA) effect. In the group of AHSS, the DSA effect was especially intensively studied in high-Mn TWIP steels [9,13,14,15]. However, the explanation is still required concerning medium-Mn steels. Some of the preliminary results on this aspect can be found in works [6,7,8,10,11,12].

Forming operations, like stamping, bending, etc., lead to an increase in temperature of processed elements due to the heat that was generated during technological operations performed at various strain rates. The temperature that occurs under operating conditions is in the range ~60–200 °C [16,17]. Some amount of heat is also generated during crash events, due to the adiabatic heating. The DSA occurs in a specific temperature range, and a value of critical strain activating the PLC effect is related to a deformation temperature. Min et al. [16] reported that the PLC effect observed in 0.2C-2Mn-1.4Si steel occurred in a temperature range of 100–250 °C. Serrations did not occur at deformation temperatures below 100 °C and higher than 250 °C. A similar tendency was observed in work [12]. Jung and De Cooman [15] observed the relationship between deformation temperature and a type of serrations in 0.6C-18Mn-0-2.5Al steel. An increase in the deformation temperature also affected the intensity of TRIP effect due to a higher stability of retained austenite [18,19]. The DSA and TRIP effects occur at the same time in medium Mn-steels, thus making the analysis more advanced.

This paper concerns the effect of elevated temperatures on the deformation behavior of 5MnNb type steel. The mechanisms affecting the plastic instabilities observed in the investigated steel were discussed based on theories available in literature.

## 2. Material and Experiments 

### 2.1. Material

A hot-rolled medium manganese 0.17C-5Mn-1.5Al-0.21Si-0.20Mo-0.03Nb (wt.%) steel was studied in this work. A relatively low carbon content of the investigated steel (0.17 wt.%) is beneficial, due to the lower probability of the PLC effect and improvement in steel weldability [3,13]. The manganese content strongly affects the PLC effect. Therefore, plastic instability is less common than in high-Mn steels in the case of medium manganese steels [13,20]. Aluminum was added to prevent carbide precipitation. This addition also prevents the occurrence of the PLC effect [15]. A Nb microaddition was included to increase the strength through grain refinement and precipitation strengthening.

The investigated steel was prepared by vacuum induction melting. Subsequently, the laboratory ingots were homogenized to eliminate the segregation of chemical composition. Afterwards, they were hot-forged (in a temperature range of 1200–900 °C) to obtain flat samples with a thickness of 22 mm. The next steps included roughing rolling (four passes) and the thermomechanical rolling process. The final thermomechanical treatment consisted of three passes at a final deformation temperature of 850 °C (sheet thickness ~4.5 mm). Subsequently, the sheet was continuously cooled to the bainitic transformation temperature and isothermally treated at 400 °C within 300 s. Finally, air cooling to room temperature was applied. Detailed information concerning the processing parameters can be found in work [21].

### 2.2. Experimental Details

Tensile specimens of 4.5 mm thickness (T) with a gauge width (W) of 12.5 mm were machined from the hot-rolled sheet along a rolling direction. The uniaxial tensile tests were performed in a temperature range of 20–200 °C at a strain rate of 10^−3^ s^−1^ while using an INSTRON 1195 universal testing machine (INSTRON, Norwood, MA, USA) that was equipped with an environmental chamber. The tensile tests were performed in accordance with the requirements of the ASTM standard [22]. The mechanical properties were determined based on average values of three measurements. The types of oscillations and a critical strain required for the initiation of the PLC effect were classified based on σ–ε curves.

The microstructure of specimens at an initial state and deformed at different temperatures were analyzed while using scanning electron microscope (SEM) (FEI, Hillsboro, OR, USA) FEI Inspect F working in a secondary electron (SE) detection mode. The energy that was used for the analysis was 15 kV. Moreover, fracture surfaces of specimens were observed. A surface topography of the investigated steel at initial state and at different temperatures was characterized while using the atomic force microscope (AFM) XE-100 Park Systems (XE-100 Park Systems, Suwon, Korea). The AFM height and amplitude maps were obtained in a non-contact mode. The AFM method was applied to determine the morphological details of individual phases. The specimens for microstructural observations (SEM, AFM) were prepared while using standard metallographic procedures. They were mechanically ground with SiC paper up to 2000 grid, polished with a diamond paste, and finally etched in 5% nital. The AFM maps of the deformed specimens were obtained from the necking area. 

The measurements of the volume fraction of retained austenite were conducted by means of an Empyrean PANalytical diffractometer (PANalytical, Almelo, The Netherlands) working at a voltage of 40 kV and a current of 30 mA. X-ray investigations were carried out while using cobalt radiation with iron filter in configuration with a Pixcel detector. A phase identification was performed according to the data from International Centre for Diffraction Data ICDD. An Averbach–Cohen method was used in order to determine the amount of retained austenite depending on the deformation temperature. This method is commonly applied to determine the retained austenite in TRIP-assisted steels [17,21,23].

## 3. Results and Discussion

### 3.1. Tensile Behavior at Different Deformation Temperatures

**Figure 1:**
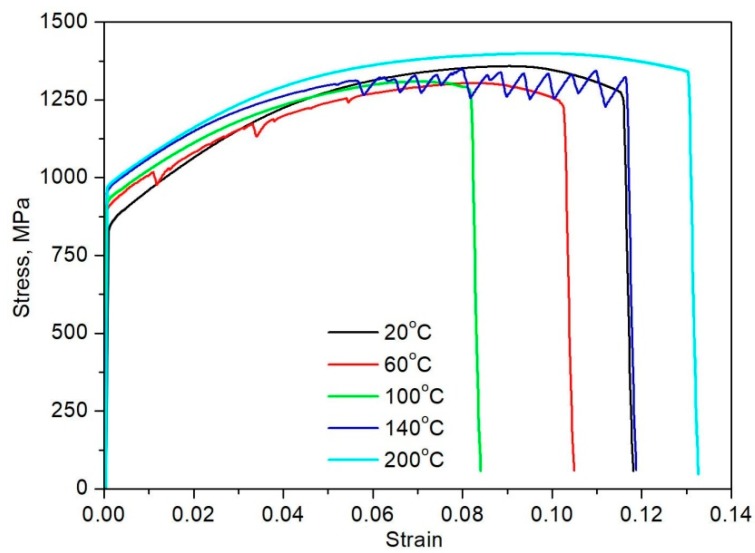
Engineering stress-strain curves of 5MnNb steel registered in a temperature range of 20–200 °C.

**Figure 2:**
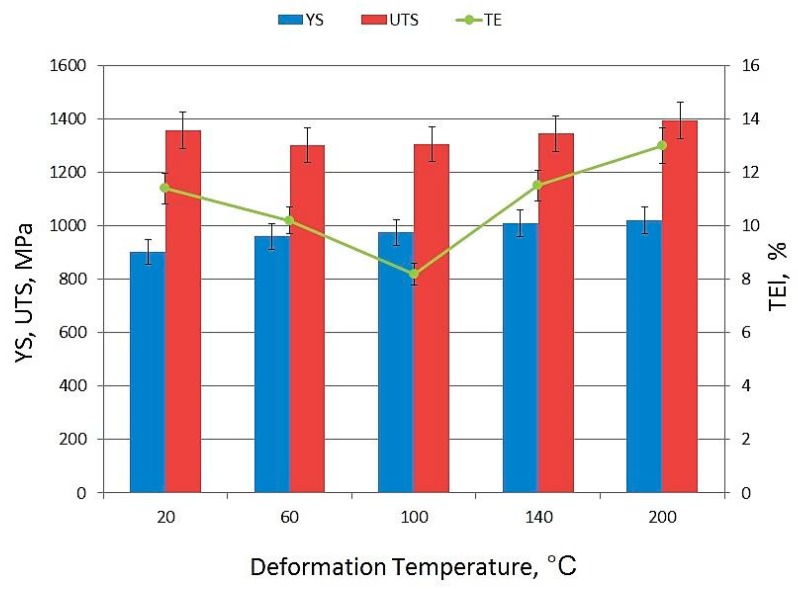
Mechanical properties of investigated steel.

Figure 1 and Figure 2 present the mechanical properties determined in static tensile tests in a temperature range of 20–200 °C. The obtained results confirm that a deformation temperature significantly affects the mechanical properties of the investigated steel and the occurrence of serrated flow phenomenon. All of the tensile curves demonstrate continuous yielding behavior without Lüders elongation (Figure 1). It is related to the presence of mobile dislocations that are generated during previous thermomechanical processing [11,20].

The TRIP steels for automotive applications should be designed to ensure the best mechanical properties in a temperature range corresponding to their exploitation and processing conditions. Ultra-high strength properties at reasonable ductility shows a specimen deformed at room temperature. UTS, YS, and TEl are: 1356 MPa, 902 MPa, and 11.5%, respectively (Figure 2). An increase in the deformation temperature to 60 °C results in a slight reduction of the mechanical properties. A smaller tensile strength and total elongation were noted in this case. A specimen deformed at 100 °C shows the lowest elongation at 8.2%. A value of total elongation was decreasing with an increase in deformation temperature in a range of 20–100 °C. Similar results were obtained by Zou et al. [24] in 0.2C-2Mn-1.6Si type steel that was deformed at quasi-static strain rates. They observed a reduction of elongation with an increase in deformation temperature in a range of 25–100 °C. Luo et al. [19] also observed some decrease in elongation in a temperature range of 20–100 °C in 0.12C-0.3Si-7Mn TRIP steel. An increase in the deformation temperature results in the reduced intensity of TRIP effect due to the higher stability of retained austenite. It is related to the increase in the stacking fault energy (SFE) of the austenite [18,25]. An increase in deformation temperature to 140 °C resulted in a further increase in the mechanical properties. The values of UTS, YS, and TEl are higher than in the specimen deformed at 100 °C. It is interesting that the highest mechanical properties were obtained for a specimen that was deformed at 200 °C. Zou et al. [24] also observed an increase of total elongation value at deformation temperatures 140 and 180 °C. They noted the increase in YS at deformation temperature in a range of 100–180 °C. The most pronounced increase in YS was observed at the highest deformation temperatures 140 and 200 °C in the case of the investigated steel.

Similar temperatures occur during the bake hardening process of automotive sheets, which is performed in a temperature range of 140–200 °C within 20 min. [26]. Similar conditions occurred during the tensile tests of samples in the present study. The samples were soaked for 30 min. before the tensile tests at 140 and 200 °C and then plastically stretched. An increase in yield strength for the specimens deformed at 140 and 200 °C was observed in comparison to the lower deformation temperatures. The same effect can be observed in the samples after bake hardening process. Thus, the continuous increase in YS in Figure 2 might be ascribed to some type of bake hardening during tensile tests. The difference is a source of dislocations that are available for the hardening. In typical BH steels, they are dislocations that are generated during pre-straining (typically 2 or 5%), whereas the dislocations are available after the previous thermomechanical processing in the investigated steel. BH effect is also related to the formation of carbides. It is thermally-activated process, thus its intensity increases with an increase in temperature. The observed increase in total elongation (Figure 2) is related to the thermal softening effect [24].

**Figure 3:**
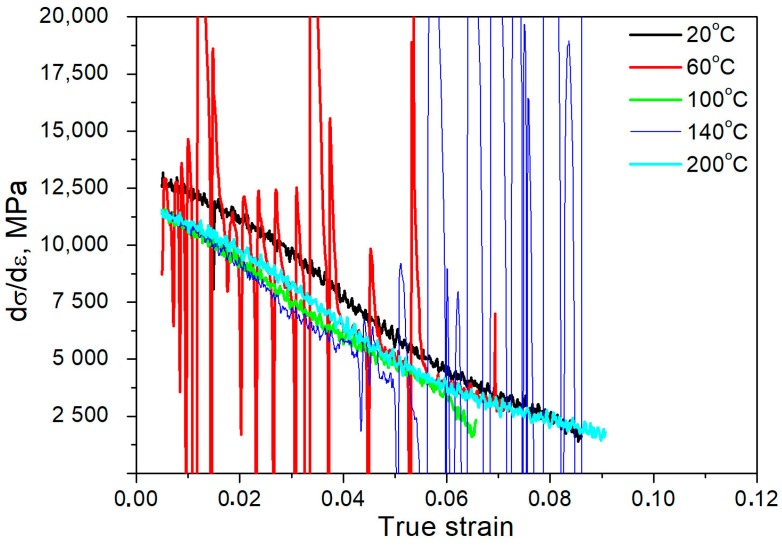
Work hardening rate as a function of true strain (in a uniform deformation range) in a temperature range of 20–200 °C.

Figure 3 shows that the deformation temperature affects the work hardening rate of the investigated steel. The dσ-dε values gradually decrease as the deformation degree increases. In steels with a large fraction of retained austenite, a local increase in dσ/dε values can be observed due to the intense TRIP effect. However, this effect was not observed for the present steels due to a relatively small austenite amount in the initial state (8.1%). The smallest slope of the curves was observed for the steel that was deformed at 20 °C. The slope of dσ-dε curves is higher for specimens deformed at elevated temperatures (60–200 °C). The different behavior of the curves that was obtained at 60 and 140 °C is related to the appearance of the PLC phenomenon. Both of the specimens show the irregular work hardening rate due to the serrations that occur in the tensile curves. The maximum work hardening rate was similar for the specimens deformed at 100, 140, and 200 °C (11,000 MPa). However, the uniform elongation was higher in the case of the steels that were deformed at 140 and 200 °C in comparison to the sample that was deformed at 100 °C. It means that some thermally activated processes are activated after the rise in temperature to 140 and 200 °C, which delay necking.

A serrated flow indicating dynamic strain aging was observed in specimens that were deformed at 60 and 140 °C (Figure 1). The specimen deformed at 20 °C shows no evidence of PLC effect. Increasing deformation temperature to 60 °C resulted in the appearance of characteristic serrations in the initial stage of plastic deformation. They were classified as a type A. A similar type and a range of serrations were observed in our previous study on similar steel without Nb microaddition [12]. An increase and then a rapid decrease in a stress value characterized the A-type of serration, while the oscillations repeat periodically [27]. A specimen deformed at 100 °C shows no serration behavior. However, characteristic oscillations were observed in a post-uniform deformation range in 0.16C-4.7Mn-1.6Al steel that was deformed at the same temperature [12]. Stable strain hardening without a serrated flow in the investigated steel is due to the lower total elongation value (Figure 2). It presumably means that a sample experienced fracture before the serrations were initiated. Increasing the deformation temperature to 140 °C resulted in pronounced intensifying of a serration amplitude and a strain range. The type B serrations were observed in this case. The B type usually occurs at elevated deformation temperatures at larger strains, while serrations regularly appear on σ–ε curves [27]. It is related to the fact that an increase in temperature enhances the mobility of solute C. Hence, carbon atoms not forming C-Mn complexes and higher strain reduce the diffusion path of the C to dislocations due to a high dislocation density. The combination of these effects could lead to a rapid recapture of dislocations by the C atoms, resulting in the observed B-type serrations [15]. Usually, evidences of plastic instability occur in a uniform elongation range. However, oscillations that were observed in 0.16C-4.7Mn-1.6Al steel [12] at the same deformation temperature were only observed in a post-uniform deformation range. The PLC effect disappeared at the highest deformation temperature 200 °C due to the accelerated carbon diffusivity.

**Figure 4:**
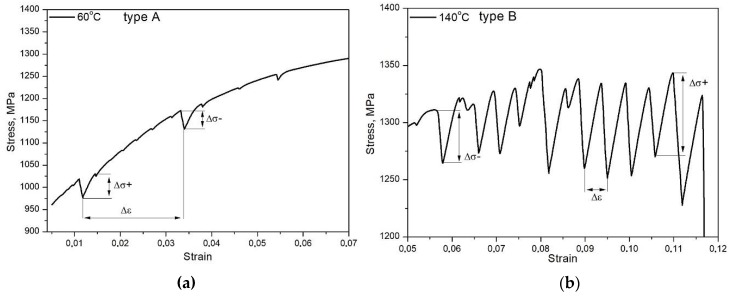
Serration features observed in steel deformed at: 60 °C (**a**) and 140 °C (**b**).

A value of the critical strain that is required for the initiation of the PLC effect is smaller in a specimen deformed at 60 °C when compared to a specimen that was deformed at 140 °C. The values of critical strain were 0.01 (Figure 4a) and 0.05 (Figure 4b), respectively. Table 1 lists detailed characteristics of serration flow at various deformation temperatures. A serration range and amplitude depend on a deformation temperature and they were higher for the sample that was deformed at 140 °C. The DSA occurs in a specific range of temperatures and a value of critical strain activating the PLC effect is related to a deformation temperature. Min et al. [16] reported that a value of critical strain in 0.2C-2Mn-1.4Si steel is higher at lower deformation temperatures. However, a reverse trend was observed in the present work and in our previous research [12] regarding plastic instability phenomenon in 5Mn steel (Figure 1). There are few theories explaining a temperature-dependent disappearance of the PLC effect. No serration behavior at 20 °C (Figure 1) is because dislocation unpinning very rapidly occurs relative to dislocation pinning. At high temperatures, where the serrations do not occur, solute diffusion is fast enough to reduce the pinning force on dislocations. The disappearance of the PLC effect at elevated deformation temperatures can be also related to carbide precipitation, which resulted in a reduced concentration of interstitial atoms in solid solution, thus terminating the DSA [28]. This theory could explain the disappearance of PLC effect in a specimen that was deformed at 200 °C (Figure 1).

The work hardening behavior of the investigated steel is related to the PLC and TRIP effects, which occur simultaneously. The PLC bands are a kind of strain localization, in which unstable austenite grains transform to martensite, due to the severe strain. However, the newly formed strain-induced martensite acts as obstacles for the propagation of PLC bands [29,30]. Sun et al. [31] noticed the localized martensite transformation occurring in the PLC areas, which spread during deformation. Similar results were noted in work [32]. They observed in the 0.2C-5Mn-2.5Al steel the TRIP effect activity and at the same time the presence of both Lüders and PLC bands. Similar results were also obtained in work [30]. Yang et al. [29] reported that the PLC bands observed in 0.3C-7Mn-2Al steel suffered strong dynamic hindrance resulting from the in-band transformed martensite during propagation and a volume fraction of the dynamically formed martensite affected the characteristics of the PLC bands. Other authors also confirmed the relationship between the PLC and TRIP effects [32,33,34,35].

### 3.2. SEM and AFM Microstructural Observations

**Figure 5:**
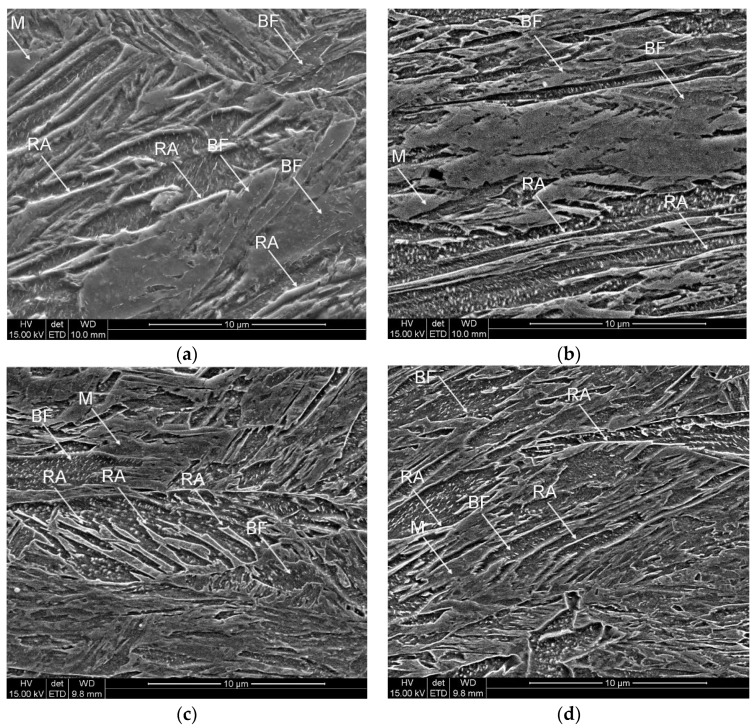
Scanning electron microscope (SEM) micrographs of the specimens: at the initial state (**a**) and deformed at different temperatures: 20 °C (**b**), 100 °C (**c**), and 200 °C (**d**).

The SEM observations were carried out to determine the microstructure of investigated steel at the initial state (non-deformed) and after tensile tests at different temperatures. Figure 5a shows the microstructure of the investigated steel in the initial state. The microstructure is composed of bainitic ferrite (BF) laths that are characterized by various thickness, some fraction of martensite (M), and retained austenite (RA). This phase can be observed as thin layers or martensitic-austenitic (MA) constituents. Some fraction of plate carbides can be observed in the thick bainitic ferrite laths. They were formed during an isothermal holding step following hot rolling. The specimens deformed at temperatures: 20, 100, and 200 °C also show the microstructures with some fraction of retained austenite (Figure 5b–d). However, most of RA changed into martensite during deformation. A small amount of RA can be observed in the form of bright, thin layers.

**Figure 6:**
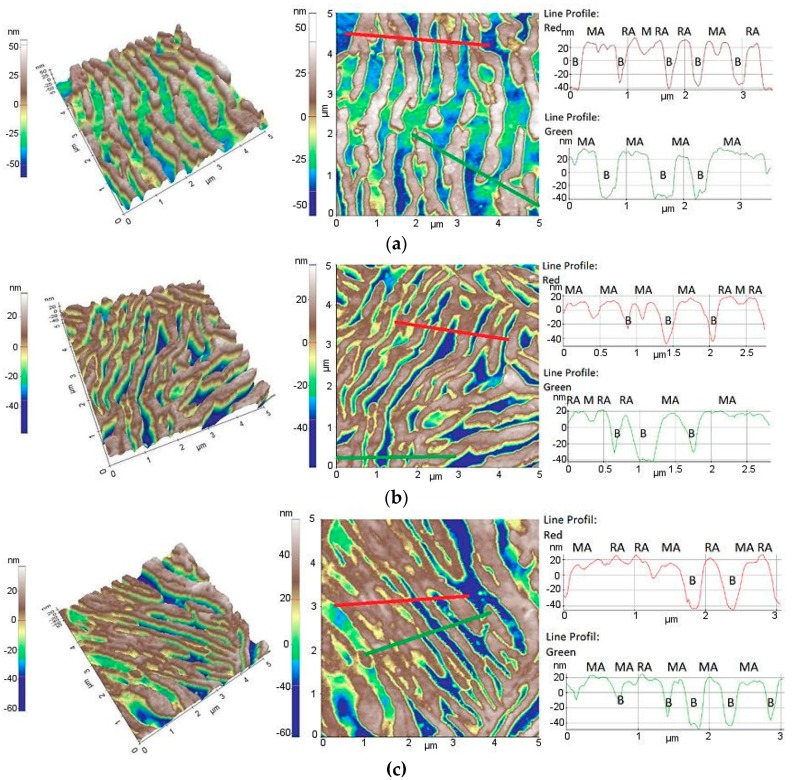
Atomic force microscope AFM maps and corresponding height profiles obtained for investigated steel at the initial state (**a**), and deformed at 100 °C (**b**) and 140 °C (**c**).

The AFM maps in Figure 6 show the surface topography of the investigated steel at the initial state and after tensile tests at different temperatures. AMF observations allowed for us to determine the location of microstructural constituents. The obtained maps indicated that the height of austenite is higher than that of bainite and martensite. Seol et al. reported the same results [36]. Evidences of retained austenite (RA), and more often martensite-austenite constituents (MA), can be observed in the investigated steel at the initial state (Figure 6a). A decrease in the height profiles within RA is characteristic for the strain-induced martensite formation. Therefore, some AFM areas that were obtained for specimens deformed at 100 °C (Figure 6b) and 140 °C (Figure 6c) show martensite (M) located between untransformed RA or more often MA topographies as martensite-austenite constituents that are located higher than the bainitic matrix (B). The freshly formed strain-induced martensite is especially located within thick austenite laths.

### 3.3. Volume Fraction of Retained Austenite

**Figure 7:**
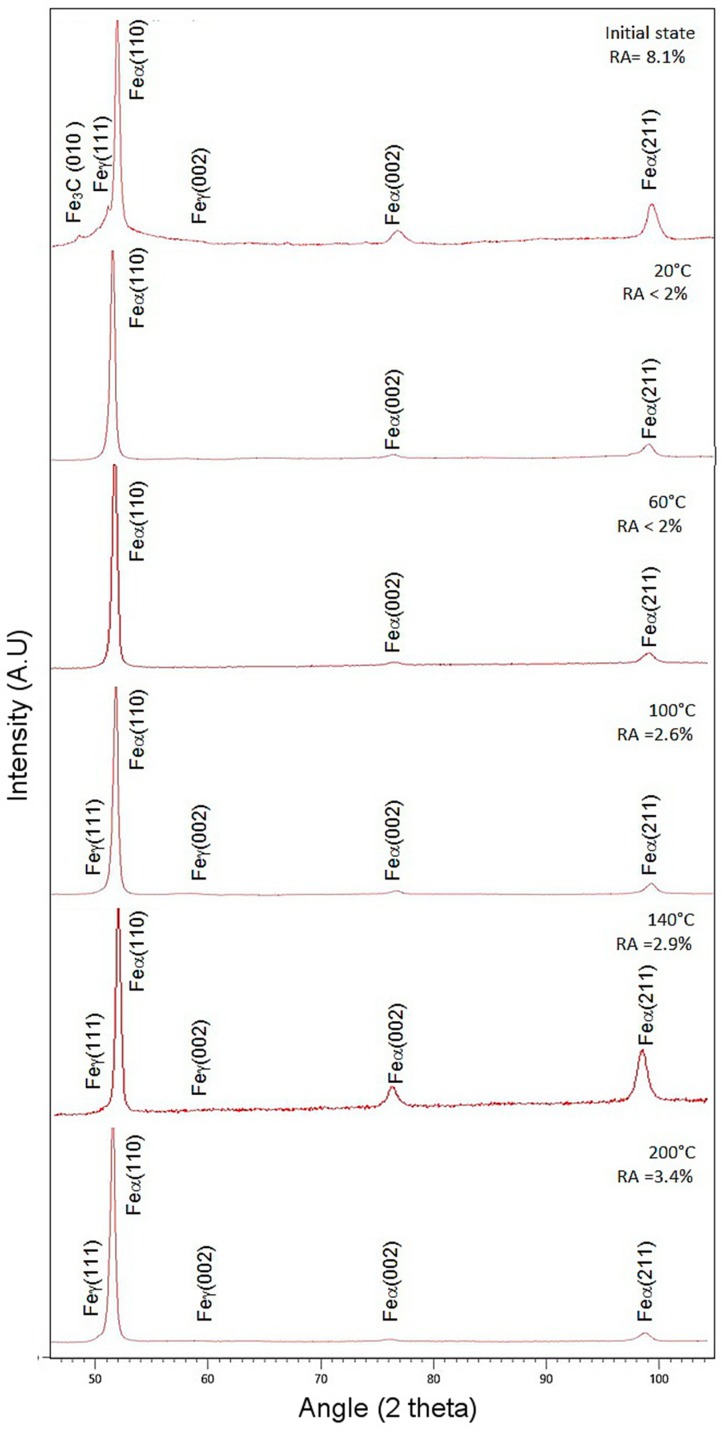
The X-Ray diffraction patterns of investigated steel at the initial state and deformed at temperatures 20–200 °C.

The XRD analysis was performed to assess the changes in a volume fraction of retained austenite, depending on the deformation temperature. In the specimen at the initial state, the presence of diffraction lines corresponding to α, γ phases and cementite was identified (Figure 7). The initial amount of RA in the investigated steel was relatively small, at 8.1%. It is related to the manganese content (5 wt.%), which affects a carbon content level in the retained austenite. As the manganese content increases the carbon content in the γ phase decreases [21,37]. Hence, the MA constituents are formed during cooling instead of austenite stabilization.

The highest fraction of untransformed austenite was detected in the specimen that deformed at 200 °C—3.4% (Figure 7). The specimens that deformed at 100 °C and 140 °C possessed ca. 2.6–2.9% of RA. In the case of specimens that deformed at 20 and 60 °C, almost all fractions of RA transformed into martensite during straining. XRD measurements show that the amount of γ phase was lower than 2%. The results of XRD analysis showed that a volume fraction of retained austenite was increasing with an increase in deformation temperature. It was due to a gradual increase in the stability of RA with the increasing deformation temperature that was caused by the higher stacking fault energy (SFE) value [38,39,40,41]. The same tendency was also observed in multiphase steels that were composed of ferrite, bainite, and retained austenite [38,39], high-Mn steels showing a TRIP effect [40], quenching and partitioning steels [24], and cold-rolled medium-Mn steels [41].

### 3.4. Fracture Behavior 

**Figure 8:**
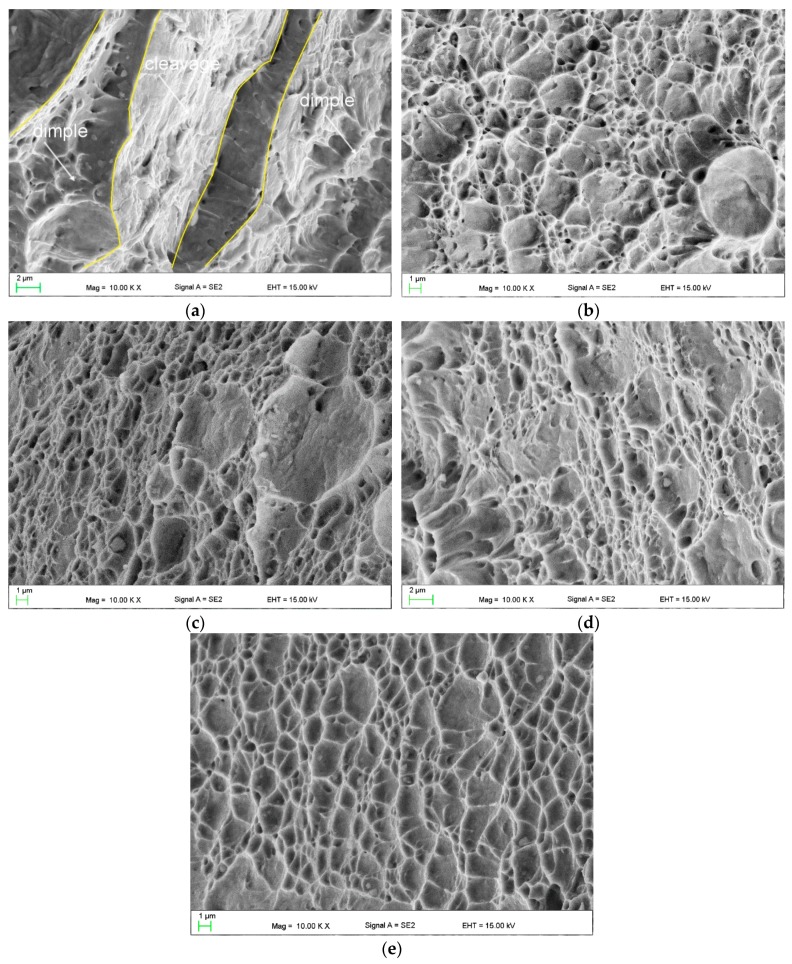
SEM fractographs of the investigated steel deformed at: 20 °C (**a**), 60 °C (**b**), 100 °C (**c**), 140 °C (**d**), and 200 °C (**e**).

Figure 8 shows the fracture surfaces of the investigated steel deformed in a temperature range of 20–200 °C. The sample that deformed at 20 °C usually contains ductile areas that are characterized by the presence of numerous dimples. However, some areas of brittle regions with a cleavage behavior are present (Figure 8a). Some microcracks can also be observed in the brittle regions. Sun et al. observed the same type of fracture showing both brittle and ductile behavior [42] in 0.2C-9.7Mn-3.2Al-3.4Si steel and by Liu et al. [43] in 0.12C-5Mn-1Al-0.2Mo-0.05Nb steel that was deformed at room temperature. The freshly formed strain-induced martensite contributes to the occurrence of cleavage-type fracture [42]. The similar behavior was also observed in DP steels [44]. Almost all fractions of retained austenite transformed into martensite in the specimen deformed at 20 °C (Figure 7). Therefore, the presence of brittle areas with terrace faults was detected in Figure 8a. The occurrence of retained austenite favors a dimple-type fracture [42]. Therefore, an increase in the deformation temperature resulting in increased stability of RA promotes the occurrence of the ductile type of fracture. The remaining austenite is more stable at elevated deformation temperatures. Thus, it can be more slowly consumed during necking, which prevents crack initiation [45]. The entirely ductile behavior occurs in a temperature range of 60–200 °C. In a range of 60–140 °C various sizes of dimples can be observed (Figure 8 b–d), whereas a very homogenous dimple size dominates at 200 °C (Figure 8e).

## 4. Conclusions 

The relationship between the Portevin–Le Chatelier effect and the deformation temperature in hot-rolled medium manganese steel was investigated. The microstructure of the investigated steel was composed of bainitic matrix, some fraction of martensite, and retained austenite dependent on a deformation temperature. The tensile curves showed the continuous yielding behavior without the yield point phenomenon and Lüders elongation. Plastic flow instabilities as characteristic serrations were observed on tensile curves in samples that were deformed at 60 °C and 140 °C. The flow stress serration of A-type was activated at 60 °C in an initial stage of deformation. A value of critical strain required for the initiation of the PLC effect, an amplitude, and range of B-type serration at 140 °C were higher than those that were observed at 60 °C. The PLC effect disappeared at the highest deformation temperature of 200 °C due to accelerated carbon diffusivity. An increase in the deformation temperature affected mechanical properties. The favorable combination of strength and ductility was noted for the specimen that was deformed at 20 °C due to the gradual strain-induced martensite transformation. The most beneficial mechanical properties were obtained at temperatures of 140 °C and 200 °C due to the occurrence of thermal softening and some fraction of untransformed austenite. The specimens that deformed at elevated temperatures showed the ductile fracture behavior, whereas some brittle regions were detected at 20 °C due to martensite formation.

## Figures and Tables

**Table 1:** Detailed characteristics of serration flow at various deformation temperatures.

Deformation Temperature [°C]	ε_cr_	Δσ+_max_ [MPa]	Δσ-_max_ [MPa]	$\bar{\Delta \sigma \;+}$ [MPa]	$\bar{\Delta \sigma -}$ [MPa]	$\bar{\Delta \varepsilon }$
60	0.01	55	42	43	32	0.022
140	0.05	82	90	64	62	0.005

ε_cr_—critical strain for initiation of the PLC effect. Δσ+_max_—maximum increase of the oscillation stress. Δσ-_max_—maximum decrease of the oscillation stress. $\bar{\Delta \sigma \;+}$—mean value of the stress increase in a range of oscillations. $\bar{\Delta \sigma -}$—mean value of the stress decrease in a range of oscillations. $\bar{\Delta \varepsilon }$—mean value of the strain period between successive oscillations.
